# A New Uniaxial Tensile Model for Foam Metal/Epoxy Interpenetrated Phase Composites

**DOI:** 10.3390/polym15040812

**Published:** 2023-02-06

**Authors:** Xiaoxing Wang, Lixin Zhang, Yu Zhao, Huijian Li

**Affiliations:** 1 School of Civil Engineering and Mechanics, Yanshan University, Qinhuangdao 066004, China; 2 College of Urban Construction, Hebei Normal University of Science & Technology, Qinhuangdao 066004, China; 3 China Water Conservancy and Hydropower Eleventh Engineering Bureau Co., Zhengzhou 450001, China

**Keywords:** foam metal, interpenetrating phase composites, damage, tensile, intrinsic relationship

## Abstract

Foam metal/epoxy interpenetrating phase composite is a new type of composite material with interpenetrating continuity in composition, which exhibits different intrinsic relationships under different stress states in tension and compression, and it is necessary to study the intrinsic relationships in the tensile state in depth. A mesoscopic damage-based tensile intrinsic model is developed, and the elasto-plastic tensile intrinsic equations of the representative volume element are derived based on small deformation theory and total strain theory, as well as the assumptions of equal stress and equal strain. The tensile strengths of nickel–iron foam/epoxy interpenetrated phase composites in three different sizes and their constituent phases were measured, and it was shown in the results that the composite of three-dimensional network interpenetration with high-strength foam metal and epoxy resin formed a weak surface inside the material, and did not significantly improve the tensile strength of the composites. The tensile instantonal equations and damage instantonal equations of nickel–iron foam/epoxy interpenetrated phase composites were predicted by the method of inversion, and the applicability and high accuracy of the tensile intrinsic model were verified in comparison with the measured results.

## 1. Introduction

Interpenetrating phase composites (IPC) are defined as multiphase composites consisting of topologically interpenetrating structures in which each phase of the material is continuous with each other. Foam metal/epoxy IPC can be designed as special materials with high strength, low modulus, and high damping [1]. The designability and interpenetrating combination of this material provides it with unique mechanical properties and desirable physical properties, and it is widely used in aviation, aerospace, transportation, energy, construction, and other fields, such as impact-resistant materials, damping materials, electromagnetic shielding materials, and phase change materials [2,3,4,5,6,7,8].

Theoretical models of the material have been studied extensively by scholars, and most of these models employ trusses to represent the reinforced phase and the rest to represent the matrix phase. Tuchinskii [9] proposed a cubic representative volume element for obtaining the thermal conductivity of bimetallic IPC, which was developed to specifically calculate the elastic constant boundary of such bimetallic IPC. Ravichandran [10] studied the deformation and creep of bimetallic IPC formed by two metals with similar properties from a microscopic perspective using a representative volume element. Feng [11] developed a model for calculating the effective elastic properties of the multiple IPC using the Mori–Tanaka method with the concept of connectivity. Yu [3,12] proposed a representative volume element with intact spheres embedded in an orthocubic unit to evaluate the material mechanical properties of foamed aluminum/epoxy IPC under the excitation of static compression and dynamic compression. Wegner and Gibson [13,14] suggested a square, triangular prismatic cell to describe bimetallic IPC and calculated its effective modulus and thermal expansion coefficient by finite elements. Seetoh et al. [15] simulated 3D printed Al_2_O_3_/Al polydimethylsiloxane IPC and Al_2_O_3_/Ni polydimethylsiloxane IPC by employing octagonal cells and Kelvin cells, calculated the compression and bending effects with COMSOL Multiphysics software and compared them with experimental results. Yuan et al. [16] proposed a finite element model of decahedra (six orthotetragonal and eight orthohexagonal) to simulate foam aluminum prisms in foam aluminum/polymer (polypropylene and acetal) IPC and calculated the strength of IPC under tensile loading. Chaturvedi et al. [17] also studied foam nickel/rubber IPC by the decahedral finite element model.

Some scholars have also studied the theory of IPC by methods such as stochastic theory, phase field theory, statistical correlation functions from a microscopic perspective, and creep equations, which usually require the assistance of finite element programs. Poniznik [18] investigated the elastic modulus, shear modulus, and Poisson’s ratio by the finite element method for interpenetrating composites in which both phases are isotropic. Xie [19] established the relationship between displacement and evolution time as a function of the Cahn–Hilliard equation for copper/ceramic IPC and studied the elasto-plastic deformation of IPC by solving this phase field equation with a computer program. Agarwal et al. [20] simulated the elasto-plastic mechanical behavior of IPC with the finite element theory of the meshless Carliogin method (EFGM). Torquato [21] proposed to derive multi-point bounds for the effective modulus of inhomogeneous materials with the statistical correlation function and discussed the effect of phase microstructure on the effective elastic properties of IPC. Basirat et al. [22] developed a microscopic model based on the Orowan creep equation to study the creep deformation of Mn-Cr bimetallic IPC.

Researchers have been following the hybrid theory of granular and laminated composites; these theories include the Voigt–Reuss upper and lower limit theories [23], based on the linear elasticity theory for particle-intercalated composites, and the Hashin–Shtrikman upper and lower limit theories [24], based on the variational principle in the linear elasticity theory for fiber-intercalated composites, as well as their modifications. Although the foam metal/polymer materials exhibit more homogeneous mechanical properties on a macroscopic scale, however, it was shown that these materials display different damage patterns under different load excitations in tension and compression in experiments [25,26], and the study with the inclusions theory was shown to have its limitations for two interpenetrating materials with large differences in mechanical properties [27].

It is a new research method to establish the equilibrium equations inside the representative volume element of two-phase material with the assumption of equal stress and equal strain at the mesoscopic scale and thereby study the tensile and compressive intrinsic equations of the representative volume elements. The authors have already presented the compressive model in the literature [28]. It is proposed to construct a mesoscopic representative volume element to characterize the intrinsic relationship of the tensile state for the foam metal/epoxy IPC in terms of the constitutive phase geometric parameters and mechanical properties derived from the damage viewpoint in this paper.

## 2. Tensile Mesoscopic Model

### 2.1. Subsection

**Figure 1:**
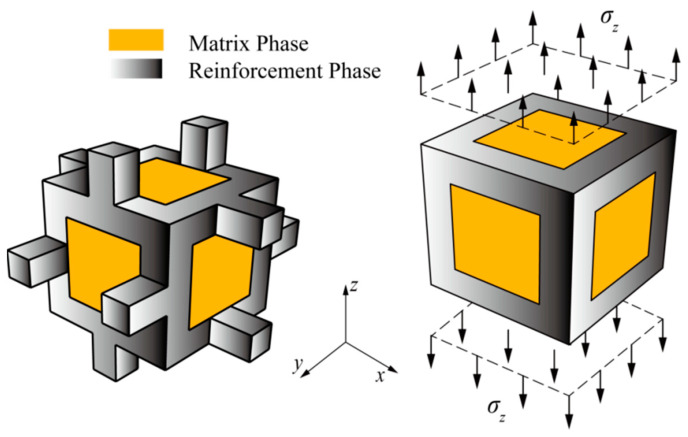
The computational model for representative volume elements and uniaxial tensile of metal/polymer IPC.

In order to obtain a simple as well as relatively accurate intrinsic equation and to facilitate the continuity of the unit model stacking at the mesoscopic level, the stretching representative volume element is adopted as a positive hexahedral geometry model, as shown in Figure 1. The peripheral prism of the orthohexahedron is represented by the reinforcement phase foam metal, and the other parts are represented by the matrix phase epoxy resin. The side length of the representative volume element is defined by the pore size of the foam metal, and the volume of the prism in the representative volume element is determined by the volume fraction of the foam metal. When defining the porous material volume fraction as the ratio of the volume of the prism of the porous material to the total volume of the unit, obviously, its value is also equal to the ratio of the density of the porous material apparent to the density of the material. Then the following equation holds:

(1) $$\frac{25.4}{n}=L+2t$$

(2) $$f=\frac{\rho^{*}}{\rho_{s}}=\frac{12L\times t^{2}+8t^{3}}{{\left(L+2t\right)}^{2}}$$

Among them, n is the pores per linear inch (PPI), $\rho^{*}$ denotes the porous density of the reinforcement phase, and $\rho_{s}$ is the density of the reinforcement phase. f is the porous material volume fraction.

**Figure 2:**
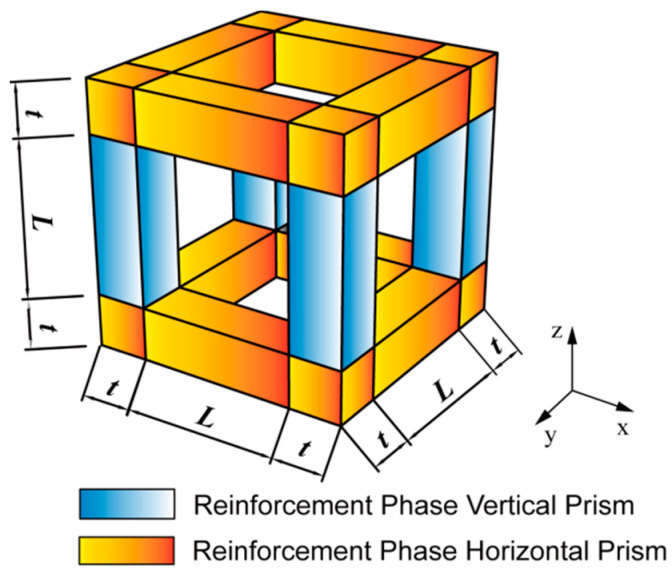
Dimensional diagram of the reinforcing phase prisms in representative volume elements of metal/polymer IPC.

To simplify the calculation, the plane shape of the prism is defined as a square, representative volume element reinforcement phase prism size, as shown in Figure 2.

A Cartesian coordinate system is established at the center of the representative volume element, and the following assumptions are applied to its tensile mechanical behavior:

1. The representative volume element is isotropic in mechanical properties; the matrix phase damage is isotropic.
2. As the load is in the far-field z-direction, the direction is parallel to the direction normal to the upper surface of the representative volume element.
3. Neither the matrix phase nor the reinforced phase undergoes volume change, and Poisson’s ratio is 0.5 in the plastic deformation stage, so the plastic spherical strain is zero in the calculation. The plastic deformation follows the conditions of total strain theory, and the loading mode is simple loading with small deformation.
4. Both the reinforcement phases and matrix phases follow the von Mises yielding criterion.

### 2.2. Deterioration Process Analysis of Mechanical Properties under Tensile Loading

It is shown that the interfacial strength in foam metal/epoxy IPC is weaker compared to the strength of each constituent phase in the experimental results [25,28,29], where cracks arise and develop from the interface of the two-phase materials first. It is assumed that the cracks in the representative volume element are distinguished into vertical and horizontal cracks according to the location of the reinforcement phase and that the principle of equal strain and equal stress is observed for each constituent phase of the foam metal/epoxy IPC during the whole tensile process.

In the early stage of tensile deformation, the stresses of the reinforcement phase and the matrix phase are small, and the deformation of each phase is consistent at the vertical prism interface of the reinforcement phase. As the load increasing, the stress of the reinforcement phase with higher elastic modulus increases significantly. When the stress difference between the two constituent phases exceeds the tangential adhesive stress at the interface, vertical cracks are generated, and the initial damage of each constituent phase material is transformed into process damage. Since the cracks exist only at the vertical interface parallel to the load direction, they have little effect on the tensile bearing capacity of the entire representative volume element.

As the load continues to increase, the stress difference between the matrix phase and the reinforcement phase exceeds the normal adhesive stress at the interface, cracks are generated at the interface of the horizontal prism of the reinforcement phase, and the crack length is identical to the prism length. It is determined by the fracture toughness of the composite material, and the stress value of the constituent phase material whether the crack extends or not after the crack is generated. If the stress strength factor determined by the stress value does not exceed the composite fracture toughness, cracks are not developed; correspondingly, vertical cracks and horizontal cracks exist at the same time in the composite element.

As the load is continued to increase when the stress strength factor reaches the composite fracture toughness, it is in a critical state, and the small stress increase causes the crack to rapidly expand to the whole cross-section, which leads to the loss of load-bearing capacity of the whole composite material, this phenomenon often occurs in the horizontal prism less cross-section, and the material shows the plastic deformation characteristics.

**Figure 3:**
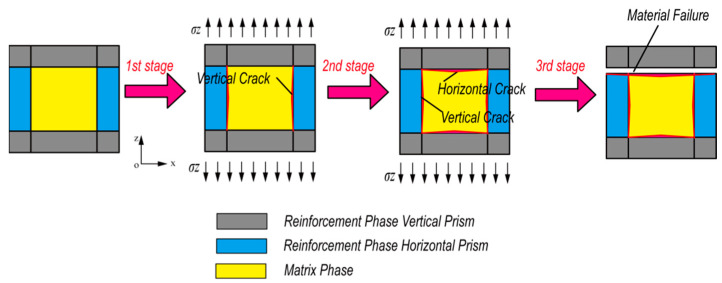
Process of tensile damage of the representative volume element.

The tensile process of the foam metal/epoxy IPC is divided into three stages according to the state of cracks in the representative volume element, as shown in Figure 3. In the first stage, the vertical cracks at the interface are completely disengaged, and matrix phase damage is generated; in the second stage, the horizontal cracks at the interface with the length of the horizontal prism are completely disengaged, and matrix phase damage develops; in the third stage, the fracture strength factor exceeds the fracture toughness of the composite, and the horizontal cracks are penetrated, causing composite failure.

### 2.3. Damage Variables

In the constituent phases of the foam metal/epoxy IPC, the effect of the metal smelting process with impurities on the mechanical properties of metal crystals is much smaller than the effect of defects such as air bubbles generated during laboratory preparation on epoxy resin. Likewise, the effect of defects on epoxy during the tensile process is significantly higher than the effect of impurities on metal crystals, which means that the damage of the composite is mainly determined by the matrix phase epoxy resin, so the physical quantity described as the degree of deterioration of the foam metal/epoxy IPC in the mesoscopic representative volume element is simplified to express as matrix phase damage. According to the mechanical model assumptions, the matrix phase damage is homogeneous. To simplify the study of the uniaxial tensile intrinsic relationship of the composite, the damage variables are defined as scalar quantities. By distributing the tensile external load to the matrix phase and reinforcement phase of the representative volume element in the cross-section perpendicular to the z-axis, the effective stress in the matrix phase can be defined as:

(3) $$\tilde{\sigma_{m}}=\frac{P_{m}}{\tilde{A_{m}}}$$

Among them, $\tilde{\sigma_{m}}$ is the effective stress in the matrix phase;

$P_{m}$ is the loads assigned to the matrix phase;

$\tilde{A_{m}}$ is the effective bearing area in the matrix phase.

By referring to the equation given by Broberg [30] describing the consideration of large deformation plastic damage, the damage variable is defined by the following equation:

(4) $$\omega_{m}=\ln\frac{A_{m}}{\tilde{A_{m}}}$$

Among them, $\omega_{m}$ is the damage variables of the matrix phase;

$A_{m}$ is the area of the matrix phase.

**Figure 4:**
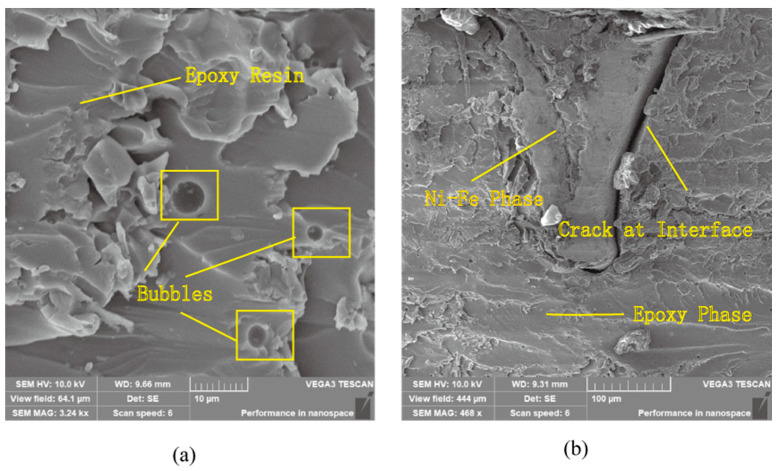
Microscopic defect diagram of Ni-Fe/Epoxy IPC tensile morphology (**a**) initial defect (**b**) process damage.

The matrix phase damage in the foam metal/epoxy IPC is expressed as two types. One is the initial damage caused during the material preparation and processing, such as the partial micro-bubbles inevitably inserted in the epoxy resin during the curing process; as shown in Figure 4a, the initial damage mainly affects the mechanical properties of the first stage of the metal/polymer IPC [31]. Another kind of damage is caused by vertical cracks and horizontal cracks and their expansion from the interface during the loading process, which mainly affects the second and third stages of the tensile state, as shown in Figure 4b. Micrographs were generated with a tungsten filament scanning electron microscope type VEGA3 manufactured by TESCAN (Czech).

## 3. Tensile Intrinsic Characterization

### 3.1. Intrinsic Equation of the Representative Volume Element

In this paper, the elasto-plastic intrinsic relationship in the tensile state of foam metal/epoxy composites is derived based on the fundamental assumption of continuum mechanics. Firstly, the mesoscopic representative volume element is topologically continuous in the component, and the intrinsic relationship of the representative volume element is used to characterize the macroscopic intrinsic relationship of the material as a whole. Secondly, the stress–strain relationship of the foam metal/epoxy IPC conformed to the generalized Hooke’s law in the elastic deformation stage and to the Ilyushin theory in the plastic deformation stage.

In the following equation symbols, the subscript m and r denotes the physical quantity of the matrix phase and the reinforcement, the subscript rV denotes the physical quantity of the vertical prism of the reinforcement phase, and the subscript rH denotes the physical quantity of the horizontal prism of the reinforcement phase.

In the elastic deformation stage, the effective stress $\tilde{\sigma_{m}}$ is used instead of the Cauchy stress, where:

(5) $${\tilde{\sigma }}_{m}=\sigma_{m}\exp(\omega_{m})$$

According to the generalized Hooke law, each constituent phase has the following equation:

(6) $${\tilde{\sigma }}_{m_{ij}}=2G_{m}\varepsilon_{m_{ij}}+\lambda_{m}\theta_{m}\delta_{ij}$$

(7) $$\sigma_{r_{ij}}=2G_{r}\varepsilon_{r_{ij}}+\lambda_{r}\theta_{r}\delta_{ij}$$

Among them, G,λ are the Lame constants. θ is volumetric strain. According to the principle of equal strain:

(8) $$\varepsilon_{m_{ij}}=\varepsilon_{r_{ij}}$$

According to the principle of equal stress:

(9) $${\tilde{\sigma }}_{m_{ij}}A_{m_{ij}}+\sigma_{rV_{ij}}A_{rV_{ij}}=T_{ij}A_{ij}$$

A is cross-sectional area. In simultaneous Equations (5)–(9), the strain in the matrix is equivalent to the average strain in the element; then the tensile intrinsic equation in the elastic stage is:

(10) $$\sigma_{ij}=T_{ij}\varepsilon_{m_{ij}}{}^{-1}\varepsilon_{ij}$$

T is the uniform external load. According to the previous assumptions, the Mises yield law is followed for each constituent phase of the material; when $\sqrt{3J_{2rV}\;}\geq \sigma_{\mathrm{sr}}$, the reinforced phase vertical prism are entered into the plastic deformation stage, and $\sigma_{\mathrm{sr}}$ is the yield strength of the reinforcement phase.

(11) $$J_{2rV}\;=\frac{1}{6}[{\left(\sigma_{rV1}-\sigma_{rV2}\right)}^{2}+{\left(\sigma_{rV2}-\sigma_{rV3}\right)}^{2}+{\left(\sigma_{rV3}-\sigma_{rV1}\right)}^{2}]$$

When $\sqrt{3J_{2m}\;}\geq \sigma_{\mathrm{sm}}$, the matrix phase is entered into the plastic deformation stage, and $\sigma_{\mathrm{sm}}$ is the yield strength of the matrix phase.

(12) $$J_{2m}\;=\frac{1}{6}[{\left(\sigma_{m1}-\sigma_{m2}\right)}^{2}+{\left(\sigma_{m2}-\sigma_{m3}\right)}^{2}+{\left(\sigma_{m3}-\sigma_{m1}\right)}^{2}]$$

In the plastic deformation stage,

(13) $${\tilde{S}}_{m}=S_{m}\exp(\omega_{m})$$

S is plastic stress bias. According to the Ilyushin formula, the following equation is established:

(14) $$\varepsilon_{m_{ij}}^{p}=e_{m_{ij}}=\frac{3}{2G_{m}}S_{m_{ij}}$$

(15) $$\varepsilon_{rij}^{p}=e_{r_{ij}}=\frac{3}{2G_{r}}S_{r_{ij}}$$

According to the principle of equal strain:

(16) $$\varepsilon_{m_{ij}}^{e}+\varepsilon_{m_{ij}}^{p}=\varepsilon_{r_{ij}}^{e}+\varepsilon_{r_{rj}}^{p}$$

According to the principle of equal stress:

(17) $$({\tilde{\sigma }}_{Hm}+{\tilde{S}}_{m_{ij}})A_{m_{ij}}+(\sigma_{Hr}+S_{rV_{ij}})A_{rV_{ij}}=T_{ij}A_{ij}$$

Among them, ${\tilde{\sigma }}_{Hm}$ is the hydrostatic pressure in the matrix phase, and $\sigma_{Hr}$ is the hydrostatic pressure in the reinforcement phase. In simultaneous Equations (13)–(17), the tensile intrinsic equation in the plastic stage:

(18) $$\sigma_{ij}=T_{ij}\varepsilon_{m_{ij}}{}^{-1}\varepsilon_{ij}$$

### 3.2. Damage Evolution Equation

In order to describe the deterioration of the properties of composite materials under loading, damage variables are brought into the model, and the corresponding damage evolution equation represents the evolutionary properties of the damage within the material in a given environment, which is also an intrinsic relationship, and the parameters of the damage evolution equation are usually derived from a series of experiments.

Lemaitre [32] gave the form of the damage evolution equation based on the principle of irreversible thermodynamics:

(19) $$Y=\rho \frac{\partial \psi }{\partial D}$$

Among them, Y is the rate of damage energy release, ρ is the constants of the material, ψ is the thermodynamic potential function, and D is the damage variables.

Lemaitre gave the mathematical form of the plastic damage evolution equation for ductility:

(20) $$\overset{•}{\omega }=\frac{K^{2}}{2ES}\left[\frac{2}{3}\left(1+\mu \right)+3\left(1-2\mu \right){\left(\frac{\sigma_{H}}{\sigma_{eq}}\right)}^{2}\right]P^{2/M}\overset{•}{P}$$

Among them, K,M is the constants of the material, S is the temperature dependent constants, $\sigma_{H}$ is the hydrostatic pressure, P is the cumulative plastic strain, and $\sigma_{eq}$ is the von Mises equivalent stress.

For the case of proportional loading without elastic deformation:

(21) $$\omega =\frac{\omega_{c}}{\varepsilon_{R}-\varepsilon_{c}}\langle P\left[\frac{2}{3}\left(1+\mu \right)+3\left(1-2\mu \right){\left(\frac{\sigma_{H}}{\sigma_{eq}}\right)}^{2}\right]-\varepsilon_{D}\rangle$$

Among them, $\omega_{c}$ is the initial damage, $\varepsilon_{R}$ is the uniaxial plastic strain at fracture, $\varepsilon_{c}$ is the uniaxial strain at initial damage, and symbol 〈 〉 is defined as:

(22) $$\langle x\rangle =\{\begin{array}{c} x,\;\;\;\;\;\;x\;>0\;\\ 0,\;\;\;\;\;\;x\leq 0\; \end{array}\;\;\;$$

These parameters for determining the damage evolution equation of composites can be obtained from tensile experiments with a constant strain rate.

## 4. Tensile Test

### 4.1. Specimens of Epoxy Resins and IPC

**Figure 5:**
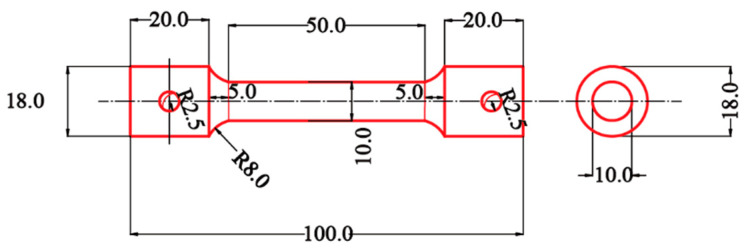
Dimensional drawing of tensile specimen(unit: mm).

Open-cell foamed Ni-Fe/epoxy IPC, a typical material with well through-porosity, was tested to verify the applicability of the tensile intrinsic structure model. There is no specific experimental standard for tensile testing of foam metal/epoxy IPC, and this paper mainly refers to the ‘Standard Test Method for Tensile Properties of Plastics’ (ASTMD638-2010) and the requirements of test equipment. The shape of the tensile specimen is similar to that of a “dumbbell”, and the specimen cross-section is circular, with the dimensions detailed in Figure 5.

**Figure 6:**
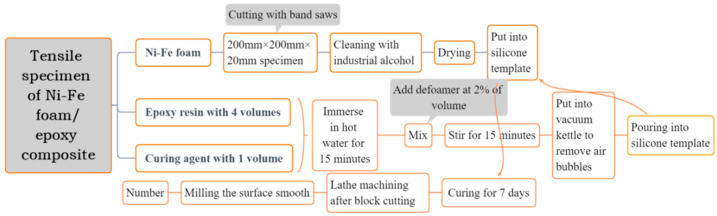
Flowchart of specimen processing.

**Figure 7:**
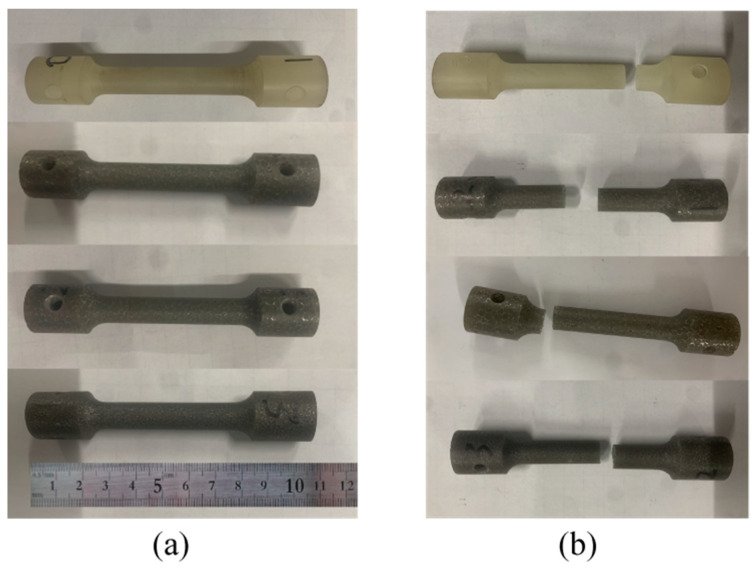
Diagram of tensile loading process of foam Ni-Fe/epoxy composite: (**a**) original specimen; (**b**) damaged specimen.

The fabrication method of the Ni-Fe/epoxy IPC specimens is shown in Figure 6, Ni-Fe/epoxy IPC specimens and epoxy resin specimens with the same size were manufactured according to Figure 5, and the machining accuracy was controlled to less than 0.1 mm. The specimens were distinguished as PPI20, PPI30, and PPI40 according to the specifications of Ni-Fe foam, and the number of each specimen was three pieces. The specimens of epoxy resin and composite materials are shown in Figure 7a.

**Figure 8:**
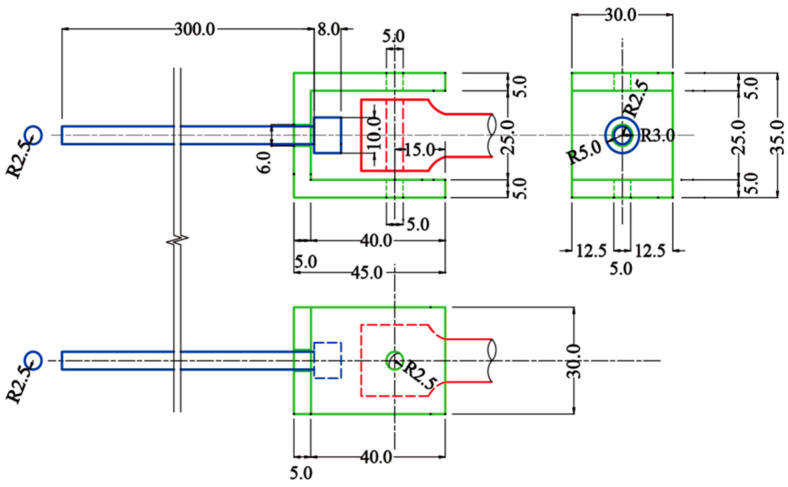
Diagram of tensile test fixture(unit: mm).

In order to avoid damage to the epoxy resin specimen and nickel–iron/epoxy IPC specimen by the chuck of the testing machine, which makes the specimen fail from its connection with the chuck and affects the accuracy of the test, a fixture was designed to fix the specimen. The fixture and the specimen were connected by pins, and both the fixture and the pins were made of Q235 steel, and the shape and dimensions of the fixture are shown in Figure 8.

### 4.2. Test Instruments and Methods

The ambient temperature of the test was controlled as the standard temperature at 25 °C. The tensile test instrument was WDW3100 micro-controlled electronic testing machine manufactured by INSTRON CORPORATION (Boston, MA, USA); the load capacity of the test machine was ±300 kN, which was able to accommodate the maximum range of experimental load requirements. The experimental data were collected automatically by computer. The test was performed by quasi-static displacement loading with a loading rate of 1 mm/min. The material properties of Ni-Fe foam are shown in Table 1.

### 4.3. Test Results and Analysis

There was no significant deformation of the failed specimens subjected to tensile loading with constant rate displacement compared to the original specimens. The specimens failed instantly after reaching the ultimate load, and the disconnection position was mostly located in the middle of the specimen. Individual specimens failed at the end of the specimen because the centers of the upper and lower collets were not fully aligned during loading, which caused secondary stresses and thus made the end of the specimen weak. The failed specimen is shown in Figure 7b.

**Figure 9:**
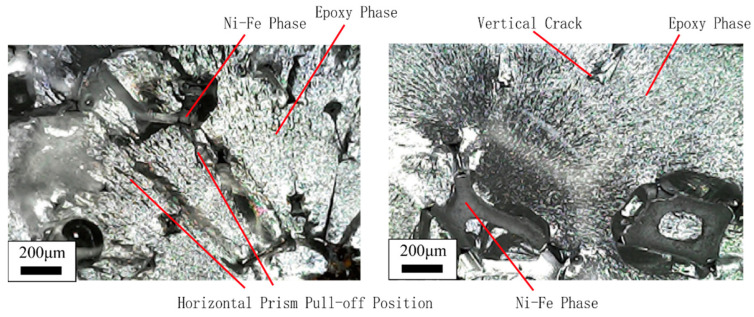
The micrograph of damage specimen in Ni-Fe/EP IPC fracture section.

The fracture sections of the failed Ni-Fe/epoxy IPC specimens were approximately flat. The cracks around the vertical prism of the PPI20 composite were slightly wider than those of the other two specifications and were easier to observe. There are some traces of horizontal prism pull-off locations, from which it can be assumed that the cracks occur first at the two-phase interface. Some of the fractured Ni-Fe phases showed necking down, and some of the Ni-Fe prisms were carried out of the transverse plane, which was more obviously observed for the PPI20 composites. The epoxy phase fracture surfaces are all relatively rough, and the micrographs of the fractured cross-sections are shown in Figure 9. Micrographs were generated with the MS5 digital microscope manufactured by RIEVBCAU (China).

**Figure 10:**
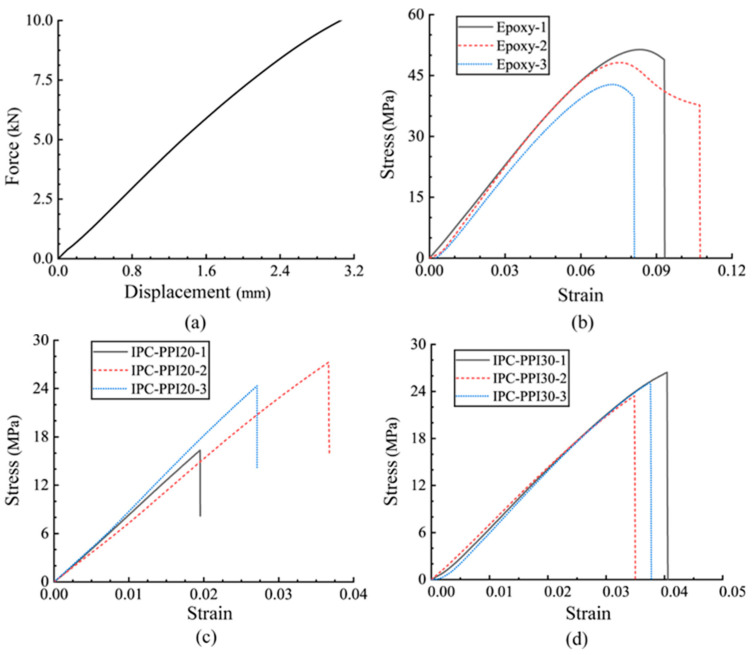

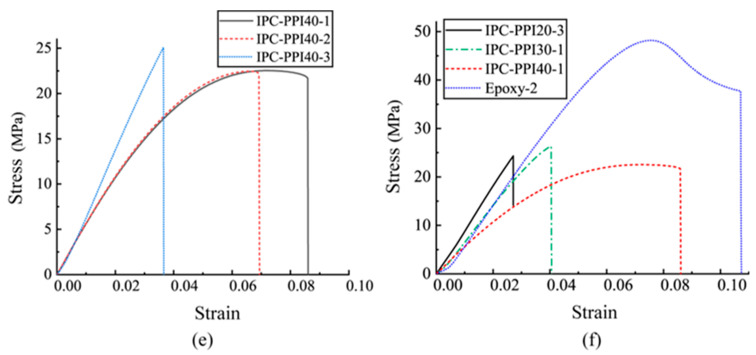
Stress–strain curve of tensile specimen: (**a**) fixture force-displacement curve; (**b**) epoxy resin; (**c**) composite PPI20; (**d**) composite PPI30; (**e**) composite PPI40; (**f**) comparison of epoxy and composites.

At the standard temperature, the fixture shows linear elastic deformation characteristics in the loading range of the tensile test, and its force–deformation curve is shown in Figure 10a. The true tensile displacement was first calculated for the epoxy resin and Ni-Fe/epoxy IPC, i.e., the measured displacement of the specimen subtracted from the tensile displacement of the fixture under equal tensile forces to obtain the true displacement of the specimen. The calculated true stress–strain curves are shown in Figure 10b–f.The epoxy resin tensile process was divided into the elastic deformation stage, plastic deformation stage, and damage failure stage, whose peak tensile stress was located at 42~48 MPa, corresponding to a strain of about 0.08, and brittle failure occurred after exceeding the peak strength, with some discrete ultimate strain.

The specimens of PPI20 Ni-Fe/epoxy composite underwent brittle fracture at the elastic deformation stage with large dispersions in peak tensile stress and ultimate strain. The strength data of specimens 2# and 3# are similar at about 24 Mpa. The reason is mainly due to the different content of partially invalid prisms in its constituent phase, Ni-Fe foam, and these initial defects make the differences in ultimate stress and strain more significant when the specimen size is small. The specimens of PPI30 and PPI40 Ni-Fe/epoxy IPC displayed some plastic deformation characteristics. The peak stress of PPI30 Ni-Fe/epoxy IPC was about 25 MPa, and corresponding to peak strain was around 0.038. The mechanical performance was quite similar for the three groups of specimens. The mechanical performance of the PPI40-1 Ni-Fe/epoxy IPC specimen was different from the other two, and we think that this performance was probably caused by material defects or handling errors, which is an incorrect data set and should be removed from the sample. The other two PPI40 IPC specimens showed typical plastic deformation characteristics; their peak strengths up to about 22 MPa were slightly lower than those of the PPI20 and PPI30 IPC specimens but exhibited more obvious plastic deformation with ultimate strains at 0.068 and 0.086. It was shown that the strength of the composite specimens was not positively correlated with their PPI values of nickel–iron foam from the comparative diagram in Figure 10f. The ultimate strain of the PPI40 composite specimens is similar to that of the epoxy specimens, but the peak stress is less than that of the epoxy specimens. It was shown that the three-dimensional network of high-strength nickel–iron alloy was interpenetrated with epoxy resin, and this combination did not enhance the tensile strength of the composites but generated a weak surface of the three-dimensional network at the interface, which reduced their tensile strength instead.

### 4.4. Tensile Testing of Ni-Fe Alloys

**Figure 11:**
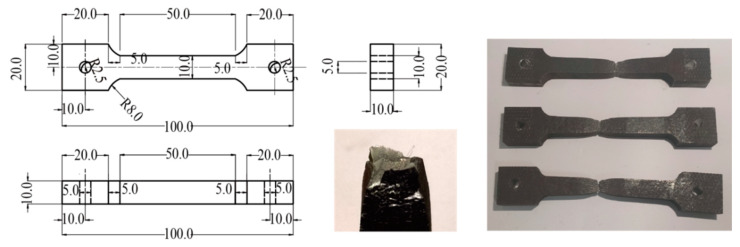
Ni-Fe tensile specimen dimensions and damage specimen diagram(unit: mm).

The tensile test of Ni-Fe alloy was referenced to ‘Standard Test Methods for Tension Testing of Metallic Materials’ (ASTM E8-04). The specimen dimensions and failure specimens are shown in Figure 11. The test piece cross-section was square size 10 mm × 10 mm, and the length of the tensile deformation section was 50 mm. The specimen was broken down in the middle during the stretching process and showed a significant necking down with a maximum elongation of about 20 mm. The fracture cross-section was jagged and displayed a concentration of stress at the corners of the cross-section, showing the shape of two opposite corners protruding and the remaining two opposite corners recessed.

**Figure 12:**
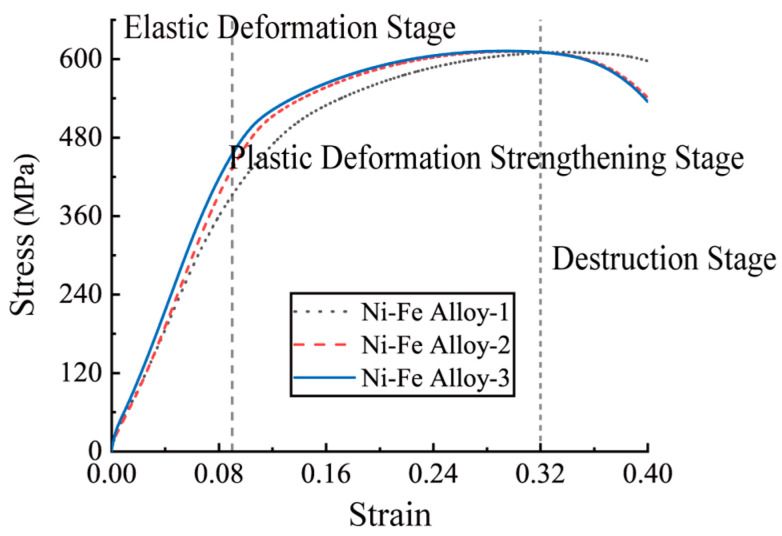
Stress–strain curve of Ni-Fe alloy in tensile test.

The tensile process of Ni-Fe alloys was divided into three stages, the linear elastic deformation stage, the plastic deformation strengthening stage, and the destruction stage. The tensile stress–strain curves of Ni-Fe alloy are shown in Figure 12. The mechanical performance of specimens 2# and 3# was relatively consistent, while specimen 1# differed slightly from the other two specimens due to machining accuracy errors. The specified plastic tensile strength was 28 MPa, the ultimate load was 610 MPa, the ultimate strain was around 0.4, and the tensile Young’s modulus was 21,014 MPa.

## 5. Verification

**Figure 13:**
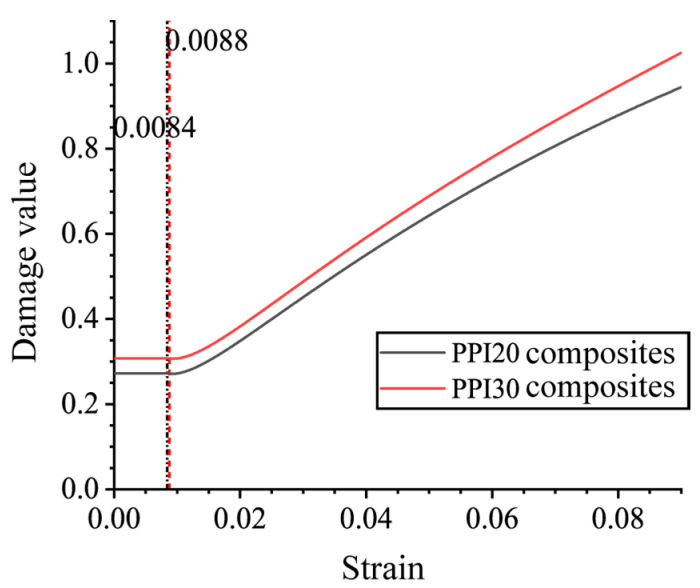
The damage intrinsic curves of PPI20 and PPI30 IPC.

The damage intrinsic equation parameters of the composites were determined by using the tensile intrinsic test curves of PPI40 Ni-Fe/epoxy IPC, and the geometry of the Ni-Fe foam prisms *L*, *t* values, and the intrinsic damage curves of PPI20 and PPI30 IPC are shown in Figure 13. By taking the damage value corresponding to the extreme low point of the curve as the initial damage value and the corresponding strain as the initial damage strain, the tensile damage intrinsic relations of PPI20 and PPI30 Ni-Fe/epoxy IPC are shown in Equations (23) and (24). The initial damage value of PPI20 IPC was found to be slightly lower than that of PPI30 IPC in the curve, which is due to the fact that the epoxy resin was cut by PPI20 Ni-Fe foam with large prism length values to form less weak interfaces in the composition of IPC.

Damage intrinsic relationship of PPI20 IPC:

(23) $$\omega =\{\begin{array}{cc} 0.27, & \varepsilon <0.0084\\ \ln\left[\frac{-0.0013+\varepsilon \left(-1.032+\varepsilon \left(-11.59+139.62\varepsilon \right)\right)}{0.00025+\varepsilon \left(-1.06+\varepsilon \left(7.52+\varepsilon \right)\right)}\right], & \varepsilon \geq 0.0084 \end{array}$$

Damage intrinsic relationship of PPI30 IPC:

(24) $$\omega =\{\begin{array}{cc} 0.31, & \varepsilon <0.0088\\ \ln\left[\frac{0.0025+\varepsilon \left(2.062+\left(23.159-278.93\varepsilon \right)\varepsilon \right)}{-0.0007+\varepsilon \left(2.075+\varepsilon \left(-15.43+\varepsilon \right)\right)}\right], & \varepsilon \geq 0.0088 \end{array}$$

**Figure 14:**
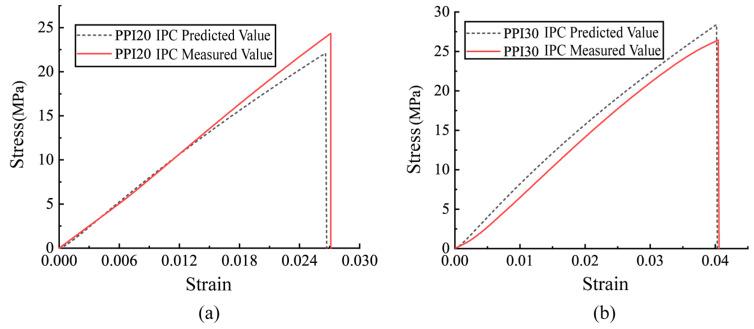
Comparison of predicted and measured results of intrinsic structure curves for Ni-Fe/EP IPC (**a**) PPI20 and (**b**) PPI30.

The tensile intrinsic equations were calculated for each of the PPI20 and PPI30 IPC in terms of the geometry of the Ni-Fe foam prisms, the damage intrinsic equation, and the intrinsic relationship of each constituent phase. The derivation process is detailed in Appendix A. Comparison of the predicted and measured stress–strain relationships for the Ni-Fe/epoxy IPC is shown in Figure 14. It was shown that the predicted results were in good agreement with the experimental data, thus verifying the reasonableness of the tensile damage intrinsic model for IPC proposed in this paper.

## 6. Conclusions

In summary, the following conclusions are offered in this paper.

1. The force characteristics of the foam metal/epoxy IPC are analyzed under uniaxial tensile loading, and a microscopic mechanical model of the tensile representative volume element for the foam metal/epoxy IPC is established. The stress–strain relationships of representative volume elements are derived for foam metal/epoxy IPC in elastic and plastic deformation phases based on the assumptions of equal stress and equal strain; the damage evolution equations of IPC are determined with the effective area of the matrix phase as the damage parameter.
2. The uniaxial tensile strengths of PPI20, PPI30, and PPI40 Ni-Fe/epoxy interpenetrated phase composites and their constituent phases (epoxy and Ni-Fe alloy) were tested in three groups each. The results show that the combination of three-dimensional networks interpenetrating does not significantly improve the tensile strength of the composites since the presence of weak interfaces.
3. The damage evolution equations of PPI20 and PPI30 IPC are determined from the measured data of PPI40 Ni-Fe/epoxy composites, geometric data, and the constitutive phase Ni-Fe and epoxy intrinsic relationships, and then the intrinsic equations of PPI20 and PPI30 Ni-Fe/epoxy IPC are predicted. Satisfactory results are obtained in comparison with experimental data, thus verifying the accuracy and applicability of the representative volume element tensile model.

## Figures and Tables

**Table 1:** Material Properties of Ni-Fe Foam.

Material Type	Apparent Density (g·cm^−3^)	Ni-Fe Alloy Density (g·cm^−3^)	Young’s Modulus of Ni-Fe Alloy (MPa)
Ni-Fe foam(PPI20)	0.18	8.23	22,847
Ni-Fe foam(PPI30)	0.21	8.23	22,847
Ni-Fe foam(PPI40)	0.26	8.23	22,847

## Data Availability

Not applicable.

## Appendix A

Equations (5) to (10) are extended to the following equations in the first stage,

(A1) $$\varepsilon_{zm}=\frac{{\tilde{\sigma }}_{zm}}{E_{m}^{e}}$$

(A2) $$\varepsilon_{zr}=\frac{\sigma_{zr}}{E_{r}^{e}}$$

(A3) $$\varepsilon_{zm}=\varepsilon_{zr}$$

(A4) $$\sigma_{zr}4t^{2}+{\tilde{\sigma }}_{zm}e^{-\omega_{0}}(4tL+L^{2})=\sigma_{z}{\left(2t+L\right)}^{2}$$

Among them, ${\tilde{\sigma }}_{zm}$ is the effective stress in the z-directional matrix phase; $\;\;\sigma_{zr}$ is the stress in the z-directional reinforcement phase; $\varepsilon_{zm},\varepsilon_{zr}$ are the strains in the z-direction matrix phase and the reinforcement phase.

To solve simultaneous Equations (A1)–(A4),

(A5) $$\varepsilon_{zm}=\varepsilon_{zr}=\frac{e^{\omega_{0}}{\left(L+2t\right)}^{2}\sigma_{z}}{4e^{\omega_{0}}E_{r}^{e}t^{2}+E_{m}^{e}L\left(L+4t\right)}$$

Then, the stress–strain equation for the Ni-Fe/epoxy IPC in the first stage:

(A6) $$\sigma =\frac{4e^{\omega_{0}}E_{r}^{e}t^{2}+E_{m}^{e}L\left(L+4t\right)}{e^{\omega_{0}}{\left(L+2t\right)}^{2}}\varepsilon$$

When $\sigma_{zr}\geq \sigma_{zm}+\sigma_{v}$, the tensile process enters the second stage, and $\sigma_{v}$ is the tangential bond stress at the interface and vertical cracks begin to appear and develop. The external load in the first stage:

(A7) $$\sigma_{z}\leq \sigma_{\max1}$$

$\sigma_{\max1}$ is the upper limit of external load in the first stage.

(A8) $$\sigma_{\max1}=\left(\frac{4e^{\omega_{0}}E_{r}^{e}t^{2}+E_{m}^{e}L\left(L+4t\right)}{e^{\omega_{0}}\left(E_{r}^{e}-E_{m}^{e}\right){\left(L+2t\right)}^{2}}\right)\sigma_{v}$$

Equations (5) to (10) are extended to the following equations in the second stage:

(A9) $$\varepsilon_{zm}=\frac{{\tilde{\sigma }}_{zm}}{E_{m}^{e}}$$

(A10) $$\varepsilon_{zr}=\frac{\sigma_{zr}}{E_{r}^{e}}$$

(A11) $$\varepsilon_{zm}=\varepsilon_{zr}$$

(A12) $$\sigma_{zr}4t^{2}+{\tilde{\sigma }}_{zm}e^{-\omega_{1}}(4tL+L^{2})=\sigma_{z}{\left(2t+L\right)}^{2}$$

To solve the simultaneous Equations (A9)–(A12),

(A13) $$\varepsilon_{zm}=\varepsilon_{zr}=\frac{e^{\omega_{1}}{\left(L+2t\right)}^{2}\sigma_{z}}{4e^{\omega_{1}}E_{r}^{e}t^{2}+E_{m}^{e}L\left(L+4t\right)}$$

Then, the stress–strain equation for the Ni-Fe/epoxy IPC in the second stage:

(A14) $$\sigma =\frac{4e^{\omega_{1}}E_{r}^{e}t^{2}+E_{m}^{e}L\left(L+4t\right)}{e^{\omega_{1}}{\left(L+2t\right)}^{2}}\varepsilon$$

When $\sigma_{h}\leq {\tilde{\sigma }}_{zm}-\sigma_{zr}(4t^{2}/4tL)$, the tensile process enters the third stage. $\sigma_{h}$ is the positive bond stress at the interface and horizontal cracks begin to appear and develop. The external load in the second stage:

(A15) $$\sigma_{\min2}<\sigma_{z}\leq \sigma_{\max2}$$

$\sigma_{\min2},\sigma_{\max2}$ are the lower and upper limits of the second stage external load, respectively.

(A16) $$\sigma_{\min2}=\left(\frac{4e^{\omega_{0}}E_{r}^{e}t^{2}+E_{m}^{e}L(L+4t)}{e^{\omega_{0}}(E_{r}^{e}-E_{m}^{e}){\left(L+2t\right)}^{2}}\right)\sigma_{v}$$

(A17) $$\sigma_{\max2}=\left(\frac{L(E_{m}^{e}L^{2}+4E_{m}^{e}Lt+4e^{\omega_{1}}E_{r}^{e}t^{2})}{e^{\omega_{1}}(E_{m}^{e}L-E_{r}^{e}t){\left(L+2t\right)}^{2}}\right)\sigma_{h}$$

Equations (13) to (18) are extended to the following equations in the third stage;

(A18) $$\varepsilon_{\mathrm{zm}}=\frac{{\tilde{\sigma }}_{zm}}{E_{m}^{p}}$$

(A19) $$\varepsilon_{zr}=\frac{\sigma_{zr}}{E_{r}^{e}}$$

(A20) $$\varepsilon_{zm}=\varepsilon_{zr}$$

(A21) $$\sigma_{zr}4t^{2}+{\tilde{\sigma }}_{zm}e^{-\omega_{1}}(4tL+L^{2})=\sigma_{z}{\left(2t+L\right)}^{2}$$

To solve the simultaneous Equations (A18)–(A21),

(A22) $$\varepsilon_{zm}=\varepsilon_{zr}=\frac{e^{\omega_{1}}{\left(L+2t\right)}^{2}\sigma_{z}}{4e^{\omega_{1}}E_{r}^{e}t^{2}+E_{m}^{p}L\left(L+4t\right)}$$

Then, the stress–strain equation for the Ni-Fe/epoxy IPC in the third stage:

(A23) $$\sigma =\frac{4e^{\omega_{1}}E_{r}^{e}t^{2}+E_{m}^{p}L\left(L+4t\right)}{e^{\omega_{1}}{\left(L+2t\right)}^{2}}\varepsilon$$
